# Synergetic Toughening Effect of Carbon Nanotubes and β-Nucleating Agents on the Polypropylene Random Copolymer/Styrene-Ethylene-Butylene- Styrene Block Copolymer Blends

**DOI:** 10.3390/polym11010029

**Published:** 2018-12-26

**Authors:** Peng-Gang Ren, Jin Wang, Qian Fan, Song Yang, Zhi-Qiang Wu, Ding-Xiang Yan, Yan-Hui Chen

**Affiliations:** 1 School of Materials Science and Engineering, Xi’an University of Technology, Xi’an 710048, China; 18149219745@163.com; 2 Department of Applied Chemistry, School of Science, and Shaanxi Key Laboratory of Macromolecular Science and Technology, Northwestern Polytechnical University, Xi’an 710072, China; fanqian1211@mail.nwpu.edu.cn (Q.F.); 19802013040@163.com (S.Y.); xg1593572468@163.com (Z.-Q.W.); 3 College of Polymer Science and Engineering, Sichuan University, Chengdu 610065, China

**Keywords:** polypropylene random copolymer, styrene-ethylene-butylene-styrene block copolymer, multi-wall carbon nanotubes, β-nucleating agents, synergetic toughening

## Abstract

Polypropylene random co-polymer (PPR)/styrene-ethylene-butylene-styrene (SBS) block copolymer blends with high toughness and favorable tensile properties were successfully obtained by blending with traces of multi-wall carbon nanotubes (MWCNTs) and β-nucleating agents (β-NAs). β-NAs can effectively induce the ductile β-form crystal in the PPR matrix. Although the addition of MWCNTs was reported to be only benefit for the tensile strength of PPR and relatively disadvantageous for the toughness, the obviously synergistic toughening effect in PPR/SBS blends was found when MWCNTs and β-NAs coexisted. The notched izod impact strength of PPR/30 wt % SBS blend with MWCNTs and β-NAs increased from 11.3 to 58.9 kJ/m^2^; more than 5-fold increment compared with pure PPR. Meanwhile, the tensile strength retention of this PPR blend is still above 72.2%. The micro-morphology indicated that the MWCNTs can act as bridges between SBS particle and PPR matrix, effectively transferring the stress and absorbing impact energy among SBS particles.

## 1. Introduction

Polypropylene random co-polymer (PPR), a co-polymerized propylene with a little amount of other olefins, is widely used in packaging, film, and other applications. Since the crystallization ability of propylene sequence is disrupted by the random insertion of a small amount of olefin units, PPR exhibits lower crystallinity with respect to propylene homopolymer (PPH), but is still considered a semi-crystalline polymer. The decreased crystallinity endows PPR with satisfied comprehensive performance, such as flexibility, transparency, and toughness compared with PPH [1,2,3]. However, the glass transition temperature (*T*_g_) of PPR did not decrease significantly by the random insertion of co-units. Hence, the impact resistance of PPR is still unappeasable for some strict applications such as outdoor materials used in severe cold area [4].

Several crystalline forms exist in PP, including monoclinic α-form, trigonal β-form and orthorhombic γ-form, among which the β-form exhibits excellent toughness due to its loose stacking structure [5,6,7]. Large amounts of β-form crystallites are usually formed in the presence of β-nucleating agents (β-NAs). Therefore, blending β-NAs with PPR is widely used to improve the toughness of PPR [4,5,6,7]. Although plenty of self-toughened β-form crystallites were generated effectively in a β-nucleated blend system, the toughening effect is still less than satisfactory [8]. To further broaden the application of PPR, blending with different kinds of elastomers has been frequently adopted due to its operative convenience and high efficiency, such as blending with ethylene-octene copolymer (POE) [9], ethylene–propylene diene terpolymers (EPDM) [10], and butadiene–styrene block copolymers (SBS) [11]. However, the improvement in impact strength usually sacrifices the tensile properties of PPR blends, due to poor interfacial adhesion and weak elastomer strength. Fortunately, the rigid nano-fillers [12], such as carbon nanotube (CNT) [13,14,15,16], crystal whisker, and calcium carbonate (CaCO_3_) [17] were reported to display obvious strength enhancement effects on the polymer matrix, while being unexpectedly beneficial for their toughness. Naturally, combining two or more of above modifiers is consequentially proposed to improve the comprehensive performance of PPR.

The synergetic effects of β-NAs and elastomers on the impact performance of PPH has been proved by previous research. For example, Ma et al. [18] demonstrated that the notched impact strength of PPH markedly increased with the addition of β-NAs and EPDM. However, the most used β-NAs exhibit unsatisfactory toughening effect on PPR because the structural regularity and stereo-regularity of PPR are inferior to that of PPH. Even so, some nucleating agents were still found to be highly efficient β-NAs for PPR [19,20]. Recently, Feng et al. [21] verified that, with the introduction of high β-nucleating efficiency of CaPim and olefin block copolymer (OBC), the PPR blends showed not only great enhancement of toughness but also the brittle-ductile transition at a low OBC content. The results revealed that combining the β-NAs and other modifiers was an efficient method to improve the toughness of PPR. Unfortunately, many studies only focused on the influence of binary modifiers on the toughness of PPR. Few works considered the toughness of PPR blends affected by ternary modifiers, i.e., the coexistence of elastomers, β-NAs, and rigid nano-fillers, especially to achieve the balance of toughness and strength of the PPR [22].

In the present work, the synergetic toughening and reinforcing effect of β-NAs, elastomers, and rigid nano-fillers on the PPR was investigated. The most common styrene-ethylene-butylene-styrene (SBS) and multi-wall carbon nanotubes (MWCNTs) were chosen as the toughener and enhancer, respectively. The aryl amide compounds (TMB-5) was chosen as β-NAs, due to its high nucleating efficiency, low cost, and commercial usage. The results indicated that ternary modifiers showed a significant synergetic toughening effect on the PPR blends, especially under high temperatures. Compared with pure PPR, the impact strength of ternary modifiers (β-NA + MWCNTs + SBS)/PPR blend with 30 wt % SBS content at 20 °C increased from 11.3 to 58.9 kJ/m^2^; more than five-fold increment. Meanwhile, the retention rate of tensile strength is as high as 72.2%. The combination of β-NAs, SBS elastomers, and MWCNTs displayed an outstanding enhancing effect on the PPR matrix.

## 2. Experimental

### 2.1. Materials

The PPR was supplied by China Petroleum and Chemical Corporation (Nanjing, China), which has an ethylene content of 7.0 mol % and a melt flow rate of 3 g/10 min (230 °C, 2.16 kg), *M*_n_ = 8.5 × 10^4^ g mol^−1^, *M*_w_ = 26.3 × 10^4^ g mol^−1^, and polydispersity index (*M*_w_/*M*_n_) = 3.08. Detailed GPC (gel permeation chromatography) information of PPR can be found in Figure S1 (Supporting Information). SBS (trade name: D1101), a ternary thermoplastic rubber grafted by maleic anhydride, was purchased from Kraton Co. (Houston, TX, USA). Multi-wall carbon nanotubes (MWCNTs, trade name: TNM5) were obtained from Chengdu Organic Chemicals Co. Ltd. (Chengdu, China). Some technical parameters are as follows: Purity: >98%, OD: 20–30 nm, ID: 5–10 nm, Length: 10–30 μm, SSA: >110 m^2^/g. SEM and TEM images of TNM5 can be obtained from Figure S2 (Supporting Information). β-NAs (trade name: TMB-5) were purchased from Fine Chemical Institute of Shanxi, Taiyuan, China. 

### 2.2. Sample Preparation

The moderate amounts of PPR particles, SBS, β-NAs, and MWCNTs powders were firstly blended by extrusion (SHJ-20 twin-screw extruder) at temperature profile of 160–195 °C from the hopper (160 °C) to the die (195 °C); the screw speed is 80 r/min. After dried at 80 °C for 2 h, the extruded granules were injection-molded into the standard dumbbell samples using an injection-molding machine HTF90W1 (Ningbo Haitian Plastic Machine Group Co. LTD, Ningbo, China) at an injection temperature profile of 190 to 200 °C from the hopper to the nozzle. The component and designation of all the blends are listed in Table 1. PPR blends are coded as CxBySz. Where the letter C, B and S represent the MWCNTs, β-NAs and SBS fillers, respectively. The number x, y and z are contents of corresponding fillers. 

### 2.3. Characterization and Testing

#### 2.3.1. Differential Scanning Calorimetry

Crystallization behavior of PPR and its blends was investigated by differential scanning calorimetry on a TA Q2000 instrument (TA Co. Newcastle, DE, USA). The samples (5–6 mg) were heated from 40 to 200 °C at a heating rate of 10 °C/min and maintained at 200 °C for 5 min to eliminate thermal history, then cooled down to 40 °C at a cooling rate of 10 °C/min. The sample was protected in nitrogen atmosphere during the entire process. The collected data were fitted by TA Universal Analysis software. The crystallinity of α-crystals (*X*_α_) and β-crystals (*X*_β_) were calculated as follows:

(1) $$X_{\alpha }=\frac{\Delta H_{\alpha }}{\Delta H_{\alpha }^{\theta }}$$

(2) $$X_{\beta }=\frac{\Delta H_{\beta }}{\Delta H_{\beta }^{\theta }}$$

where Δ*H*_α_^θ^ is the melting enthalpy when the content of α-crystal is 100%, 178 J g^−1^. Δ*H*_β_^θ^ is the melting enthalpy when the content of β-crystal is 100%, 170 J g^−1^ [23], Δ*H*_α_ and Δ*H*_β_ were obtained by TA software from DSC curves.

#### 2.3.2. Scanning Electron Microscopy (SEM)

The fracture morphologies of the MWCNTs, PPR, and its blends were observed by using a field emission scanning electron microscope (FEI Nova NanoSEM 450, Hillsboro, OR, USA) after gold sputtering. To observe the phase morphology of the SBS elastomer, the samples were cryogenically fractured after keeping in liquid nitrogen for 1 h. Subsequently, the fractured samples were put in tetrahydrofuran at room temperature for 12 h to etch the SBS dispersed phase. After sputtering gold, the samples were also observed by using a field emission scanning electron microscope (FEI Nova NanoSEM 450, Hillsboro, OR, USA) under an acceleration voltage of 20 kV. 

#### 2.3.3. X-Ray Characterization

Two-dimensional wide-angle X-ray diffraction (2D-WAXD) and small-angle X-ray scattering (2D-SAXS) measurements were conducted at the beamline BL16B1 of Shanghai Synchrotron Radiation Facility (SSRF, Shanghai, China). For WAXD, the mono-chromated X-ray beam with a wavelength of 0.124 nm was focused to an area of 80 × 80 μm^2^ (length × width), and the sample-to-detector distance was set as 152.7 mm. The 2D-WAXD images were collected with an X-ray CCD detector (Model SX165, Rayonix Co. Ltd., Evanston, IL, USA.). In the SAXS measurement, the sample-to-detector distance was fixed at 1995 mm. To obtain a discernible X-ray scattering intensity, a piece of slice with width of 6 mm and thickness of 1 mm was carefully machined from the injection-molded parts. 2D-WAXD and 2D-SAXS signals come from the central part of the sample. 1D-WAXD profiles were obtained from circularly integrated intensities of the acquired 2D-WAXD patterns. Detailed information can be obtained from our previous researches [22,24,25].

1D-SAXS profiles were obtained from the circular average of 2D-SAXS patterns, in which scattering intensity was plotted as a function of the reciprocal space vector, ***s***(|***s***| = 2 sin *θ/λ*, where *λ* is the wavelength of the incident beam and 2*θ* the scattering angle). The long period (*L*_B_) that defines the statistical average of the distance between two lamellae is determined by the position of the first intensity maximum (***s****m*) in 1D-SAXS profiles by *L*_B_ = 1/***s****m* [24]. The thickness of crystalline lamella (*L*_c_) can be estimated by *L*_c_ = *X*_c_(WAXD) × *L*B, and the thickness of the confined amorphous phase *L*_a_ = *L*_B_ − *L*_c_ [26,27].

#### 2.3.4. Dynamical Mechanical Analysis

Dynamical Mechanical Analysis (DMA) was carried out with a Q800 DMA instrument (TA Instruments, Newcastle, DE, USA) at heating rate of 3 °C/min and at a frequency of 1.0 Hz. The testing range was from −100 °C to 120 °C. Storage modulus and tangent of loss angle would be obtained, while *T*_g_ was taken from the peak temperature of *tan*δ curve.

#### 2.3.5. Transmission Electron Microscope (TEM)

Transmission electron microscopy (TEM) was performed with a FEI Talos F200X (Hillsboro, OR, USA) electron microscope. Stable dispersion of the MWCNTs (dispersed in ethanol) was prepared by ultrasonic treatment. A drop of stable dispersion was placed on a copper grid and then dried before it was transferred to the sample chamber. The dispersion state of MWCNTs in polymer matrix was investigated on a ultrathin composite sample with a thickness of 100 nm, which was prepared on an Ion Beam Thinner (LEICA, RES102, Wetzlar, Germany).

#### 2.3.6. Impact Tests

The notched izod impact tests were conducted according to the standard GB/T 1843-2008 at temperatures of −20 °C, −10 °C, 0 °C, and 20 °C. At least five samples of 60 mm × 10 mm × 4 mm were tested and the average values were adopted. These samples were carefully machined with a single V-notch of 2 mm depth. The notched izod impact strength can be calculated from following equation:

(3) $$a_{iN}=\frac{E_{c}}{h\times b_{N}}{\times 10}^{3}$$

where Ec is the corrected fracture energy of specimens, J; *h* is the thickness of specimens, mm; *b*_N_ is the remaining width of specimens, mm.

#### 2.3.7. Tensile Tests 

The tensile tests were conducted according to the standard GB/T 1040-92 at temperatures of 23 °C. Dumbbell-shaped samples were used to test the tensile property on an SANS (Suns) universal testing machine (Shenzhen, China) with gage length of 100 mm at 50 mm/min. The size of the tensile sample was controlled by injection mold with 150 mm (Length) × 10 mm (Width) × 10 mm (Thickness). At least five samples were tested and the average values were adopted.

## 3. Results and Discussion

### 3.1. Notched Izod Impact Performance

**Figure 1:**
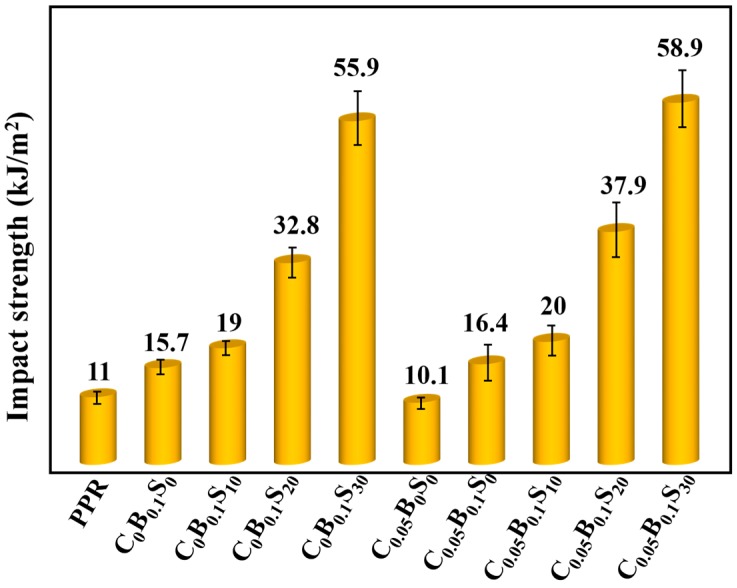
Izod impact strength of various polypropylene random co-polymer (PPR) blends (20 °C).

The notched izod impact strength of PPR and its blends with various modifiers are shown in Figure 1. The presence of β-NAs enhanced the impact strength of PPR. Notched izod impact strength of PPR increased from 11 to 15.7 kJ/m^2^ with the addition of only 0.1 wt % β-NAs, improved by 42.7%. β-NAs can induce PPR to form the ductile β-form crystallites and thus improve the impact strength of PPR [1,4,21]. Moreover, the impact strength of PPR blends markedly increased with the increasing SBS elastomer in the presence of β-NAs, especially for the high SBS loading. With the addition of 30 wt % SBS (C_0_B_0.1_S_30_), the notched izod impact strength of β-nucleated PPR increased from 15.7 to 55.9 kJ/m^2^, showing an increment of 256% than C_0_B_0.1_S_0_. Compared with C_0_B_0.1_S_30_, the sole addition of MWCNTs (C_0.05_B_0_S_0_) reduced rather than increased the impact performance of PPR. i.e., the notched izod impact strength of PPR slightly decreased from 11 to 10.1 kJ/m^2^ in the presence of traces of MWCNTs (0.05 wt %). However, the notched izod impact strength of (MWCNTs + β-NAs)/PPR and (MWCNTs + β-NAs + SBS)/PPR blend are superior to that of β-NAs/PPR and (β-NAs + SBS)/PPR at the same filler contents, respectively. For example, the notched izod impact strength of C_0_B_0.1_S_0_ increased from 15.7 to 16.4 kJ/m^2^ with traces of MWCNTs loading (C_0.05_B_0.1_S_0_). And the corresponding value of the C_0_B_0.1_S_30_ blend increased from 55.9 to 58.9 kJ/m^2^ in the presence of MWCNTs. These results suggest that the combination of rigid MWCNTs and flexible β-NAs/SBS modifiers show an obviously synergistically toughening effect on the PPR, although the sole rigid MWCNTs filler generally has a negative effect on the toughness of PPR.

**Figure 2:**
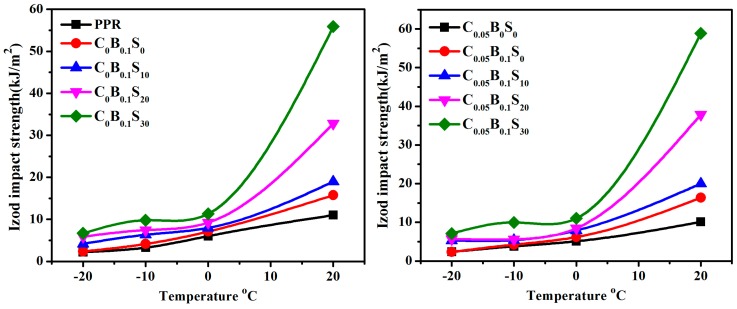
Izod impact strength of PPR and its blend at various temperature.

The notched izod impact strength of PPR, and its blends at different temperatures, are displayed in Figure 2. Detailed information of impact strength for PPR and its blends can also be found in Figure S3 (Supporting Information). The notched izod impact strength of all samples significantly increased with the increasing testing temperatures. This is mainly attributed to the volume expansion and enhancement of molecular mobility at high temperature, thus weakening the intermolecular forces. Even so, the trends of impact strength with various fillers are entirely different. The izod impact strength of pure PPR and its blend with a single modifier (i.e., β-NAs/PPR and MWCNTs/PPR) almost linearly rose with the increase in temperature. With the addition of small amounts of SBS elastomer particles (<10 wt %), the impact strength of PPR blends still exhibited a linear relationship versus temperature. When the SBS content exceeded 20 wt %, the impact strength of PPR blends started to deviate from the linear relationship with the increasing testing temperature, especially at high testing temperatures. A dramatic rise of impact strength happened when the testing temperature exceeded 20 °C. For example, when the testing temperature was raised from 0 to 20 °C, the impact strength of C_0_B_0.1_S_30_ and C_0.05_B_0.1_S_30_ increased from 11.3 and 11 kJ/m^2^ to 55.9 and 58.9 kJ/m^2^; almost four-fold and more than five-fold increments, respectively. However, the corresponding enhancements of PPR, C_0_B_0.1_S_0_ and C_0.05_B_0_S_0_ are only 83.3% (from 6 to 11kJ/m^2^), 121% (from 7.1 to 15.7 kJ/m^2^) and 98.0% (from 5.1 to 10.1 kJ/m^2^), respectively. It is also worth noting that the notched izod impact strengths of the ternary modifiers (MWCNTs + β-NAs + SBS)/PPR blends were inferior to that of the binary modifiers (β-NAs + SBS)/PPR blends at low temperature (≤0 °C). Nonetheless, it becomes reversed at a high temperature (20 °C). At this time, the ternary modifiers/PPR blends exhibited superior impact strength to binary modifiers/PPR blends (without MWCNTs). Hence, some inherent factors related to the toughness of PPR blends need to be further investigated. 

### 3.2. Tensile Properties

**Figure 3:**
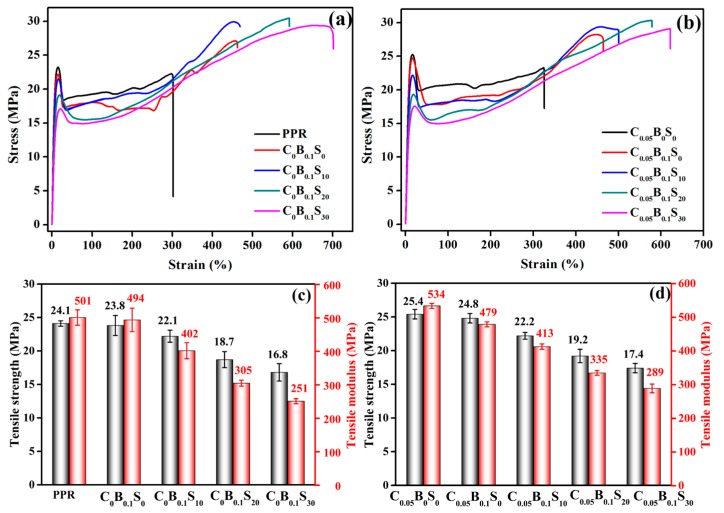
Stress-strain curves (**a**,**b**), tensile strength (**c**) and tensile modulus (**d**) of PPR and its blends.

The tensile properties of PPR and its blends, including stress-strain curves, tensile strength (*σ*), and tensile modulus (*E*) are shown in Figure 3. Compared with pure PPR, the presence of β-NAs slightly deteriorated the tensile strength and modulus of PPR, from 24.1 and 501 MPa to 23.8 and 494 MPa, respectively. While the elongation at break significantly increased by 46.2%, from 316 to 462%. It may be explained by the formation of trigonal β-form crystal induced by β-NAs, which is not as dense as monoclinic α-form crystal, but more flexible than the latter [5,6,7]. The addition of SBS remarkably decreased the tensile strength and modulus of PPR blend, but the elongation at break increased simultaneously, thus making the PPR blend more malleable. Unlike the β-NAs, MWCNT may induce the nucleation of PPR and form relatively brittle α-form crystal [25]. Combined with its excellent mechanical properties, the tensile strength and modulus of PPR were slightly improved with the addition of traces of MWCNTs. The tensile strength of the ternary modifiers/PPR blend with 30 wt % SBS contents (C_0.05_B_0.1_S_30_) was as high as 17.4 MPa, about 72.2% retention rate of pure PPR (24.1 MPa), manifesting an outstanding comprehensive enhancing effect of the ternary modifiers. 

### 3.3. Melting and Crystallization Behavior

**Figure 4:**
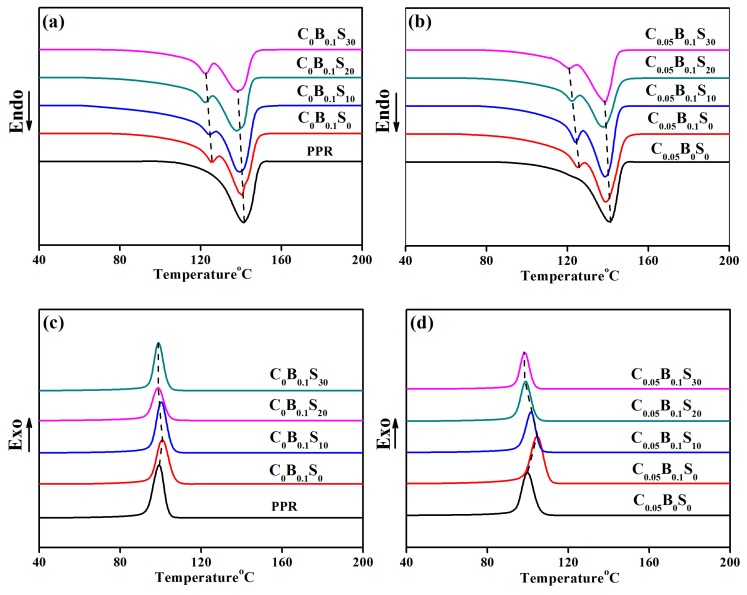
The melting (**a**,**b**) and crystallization (**c**,**d**) curves of PPR and its blends.

Since the crystallization behavior of PPR blends have a major impact on their mechanical properties, the melting and crystallization behavior of PPR and its blends were investigated, as shown in Figure 4. A strong peak at about 140 °C in all the samples represents the melting of the monoclinic α-form crystal. A small peak at about 125 °C, assigned to trigonal β-form crystal, was only observed in the PPR blends containing β-NAs modifier. These results suggested the β-NAs can effectively induce β-form nucleation in the presence of other fillers. More remarkably, the melting peaks of both α-form and β-form crystal shifted to lower temperatures with the increasing SBS contents (marked by the dotted line in Figure 4a,b). This may be explained by the restriction of crystallization behavior of PPR with the existence of SBS particles, which weakens the crystallization of both α-form and β-form crystals. Such structural changes were also observed from the crystallization curves of PPR blends (Figure 4c,d). Compared with PPR (*T*_c_ = 99.2 °C), C_0_B_0.1_S_0_ blend exhibits higher crystallization temperature (100.9 °C), indicating the enhanced crystallization effect of β-NAs on the β-form crystal of PPR. The same conclusion is also obtained from the comparison between C_0.05_B_0_S_0_ (99.7 °C) and C_0.05_B_0.1_S_0_ (104.7 °C). However, with the addition of SBS elastomer, the crystallization temperature of the PPR blends decreases. The more SBS elastomer contents, the lower crystallization temperature of PPR blends. Detailed crystallization parameters are shown in Table 2. The total crystallinity of PPR increased from 0.19 to 0.24 in the presence of β-NAs, which is attributed to the formation of about 10% β-form crystal, whereas the total crystallinity of the PPR decreased with the increased SBS contents. Interestingly, the crystallinity of β-form hardly changed whereas the crystallinity of α-form obviously decreased from 0.21 to 0.16 as the SBS content increased from 0 (C_0_B_0.1_S_0_) to 30 wt % (C_0_B_0.1_S3_0_). This further suggests that the formation of β-form crystal is greatly affected by the β-NAs, and is scarcely influenced by SBS elastomer. On the contrary, the α-form crystals exhibit a strong dependence on the contents of SBS elastomer. Furthermore, higher α-form crystallinity of C_0.05_B_0_S_0_ (0.21) compared with PPR (0.19) and C_0_B_0.1_S_0_ (0.16) indicate that the trace amount of MWCNTs are beneficial to the production of α-form crystals. However, both the crystallinity of PPR and the *X*_β_ in ternary modifiers/PPR blends decrease with the increasing SBS content, while the influence of MWCNTs on α-form crystallinity is negligible, especially at a high SBS loading (as shown in Table 2). It suggests that the trace amount of MWCNTs rarely affects the formation of α-form crystal but inhibits the formation of the β-form crystal with the presence of SBS, especially at high SBS contents. Even so, the ternary modifiers/ PPR blends still exhibit higher and higher impact strength with the increasing SBS content. It is seen here the presence of β-form crystal enhances the toughness of the PPR blends, but is not the determined factor. Taking an example, lower content of β-form crystal in C_0.05_B_0.1_S_30_ still exhibit higher impact strength compared with C_0_B_0.1_S_30_.

**Figure 5:**
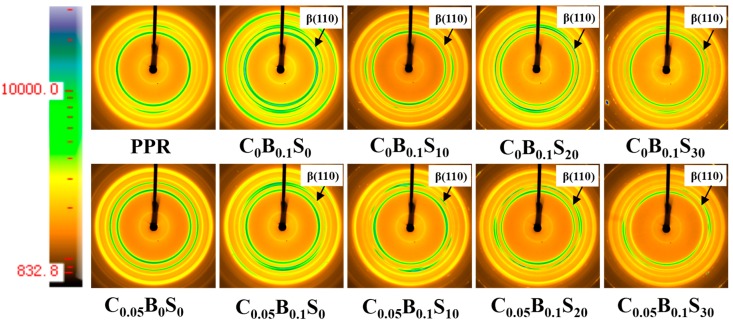
2D-WAXD patterns of the PPR and its blends (Color bar represents Diffraction Intensity).

To further explore the crystal structure and composition of PPR blends, 2D-WAXD measurement was adopted here. The 2D-WAXD patterns are shown in Figure 5. The overall crystallinity (*X*_c_), relative amount of the β-form crystal (*K*_β_) and β-crystallinity (*X*_β_) can be calculated from 1D-WAXD (as shown in Figure S4 (Supporting Information)). The *K*_β_ is obtained from Equation (4) Tuner-Jones et al. [28]:

(4) $$K_{\beta }=\frac{A_{\beta }(110)}{A_{\beta }(110)+A_{\alpha }(110)+A_{\alpha }(040)+A_{\alpha }(130)}$$

where *A*_β_ (110) represents the area of the β(110) reflection peak; *A*_α_ (110), *A*_α_ (040), and *A*_α_ (130) represent the area of the α (110), α (040), and α (130) reflection peaks, respectively. Meanwhile, the crystallinity of β-form crystal (*X*_β_) was given by Equation (5):

*X*_β_ = *K*_β_*X*_c_ (5)

The corresponding data of *K*_β_ and *X*_β_ are shown in Table 3. The crystallinity of the PPR increased slightly from 0.35 to 0.36 (C_0_B_0.1_S_0_) in the presence of β-NAs. While the total crystallinity of the PPR decreased with the SBS contents increasing. Only 0.28 crystallinity was obtained for the PPR blend with 30 wt % SBS contents (C_0_B_0.1_S_30_), but the crystallinity of β-form increased from 0.05 to 0.09. With the addition of MWCNTs, the total crystallinity of PPR and the *X*_β_ of C_0.05_B_0.1_S_0_ decreased respectively from 0.35 and 0.09 to 0.26 and 0.05 at 30 wt % SBS loading. The results are in consistence with the DSC data. In addition, the β-form crystal was only formed in the blends containing the β-NAs. This confirmed that β-NAs effectively induce PPR to form β-form crystal. 

**Figure 6:**
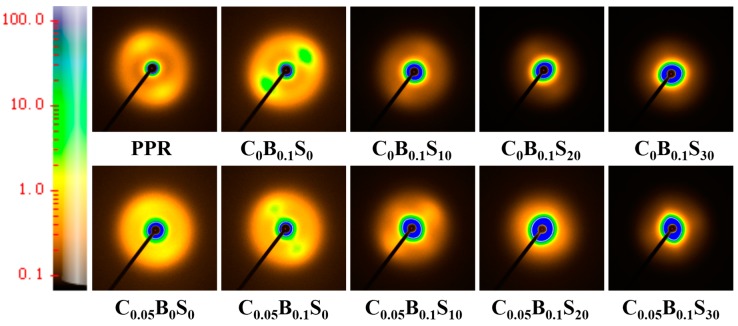
2D-SAXS patterns of the PPR and its blends (Color bar represents Scattering Intensity).

Furthermore, the crystal lamellar parameters were obtained from the 2D-SAXS measurements (as shown in Figure 6). The 1D-SAXS curves integrated from 2D-SAXS are shown in Figure S5 (Supporting Information). Detailed information including the long period (*L*_B_), the thickness of crystalline lamella (*L*_c_) and the thickness of the confined amorphous phase (*L*_a_) are listed in Table 4. As a result of the loose lamellar structures of β-crystals, all the β-nucleated PPR samples show higher *L*_B_ than the non-nucleated ones. The increase in SBS also increased *L*_a_, verifying the immersion of SBS molecular chains into PP lamellae. The presence of MWCNTs in the β-nucleated PPR/SBS blends further weakened the lamellar regularity, as evidenced by the further enlarged *L*_a_. This is also consistent with the decreased crystallinity in the β-nucleated PPR/SBS blends with MWCNTs, as shown in Table 2 and Table 3. This behavior is favorable due to the toughness of PPR/SBS blends and the enhanced mobility space of molecular chains of PPR. 

### 3.4. Phase Morphology 

**Figure 7:**
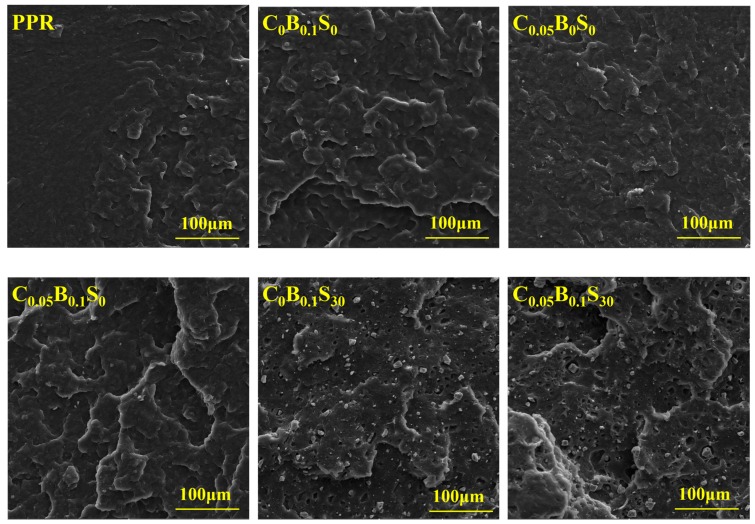
The impact fractured surface of the PPR and its blends (at 20 °C).

In order to investigate the toughening mechanism of modifiers on the PPR matrix, SEM images of PPR and its blends were obtained, as shown in Figure 7. Plastic deformation in a small area is observed in the pure PPR, displaying a typical brittle fracture. The comparatively rough fractured surface of C_0_B_0.1_S_0_ reveals that the ductile fracture occurs in the β-NAs/PPR blend due to the formation of β-form crystal induced by β-NAs. Similar to pure PPR, brittle fracture happens in the C_0.05_B_0_S_0_ blend, indicating that the addition of MWCNTs does not improve the toughness of the PPR. When the SBS elastomer particles were added, a mass of half-bare SBS elastomer particles and the subsidence holes existed in the C_0_B_0.1_S_30_ and C_0.05_B_0.1_S_30_ blends besides the irregular fracture surface. The crack deflection and the pullout of the elastomer particles occur upon impact, which are favorable for the dissipation of the impact energy.

**Figure 8:**
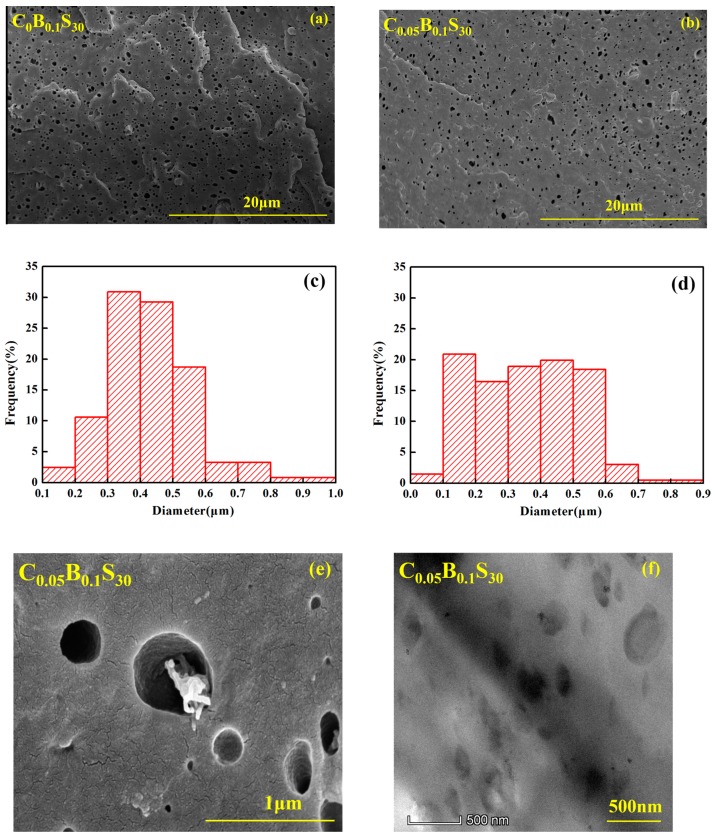
SEM micrographs of of PPR blends with 30 wt % SBS after etched bytetrahydrofuran (**a**,**b**); SBS size distribution of C_0_B_0.1_S_30_ (**c**) and C_0.05_B_0.1_S_30_ (**d**); enlarged SEM (**e**) and TEM (**f**) image of C_0.05_B_0.1_S_30_.

The SEM images of the fracture surface of PPR blends etched by tetrahydrofuran are shown in Figure 8a,b. Small and oval SBS particles appear in the PPR blend with high SBS contents, which may be caused by high shear stress during the mixing process as a result of the increased viscosity of matrix. Figure 8c,d showed that the average size of the SBS particles is as small as about 0.3–0.5 µm in both C_0_B_0.1_S_30_ and C_0.05_B_0.1_S_30_. Compared with that of C_0_B_0.1_S_30_, the SBS particles are more uniformly distributed in C_0.05_B_0.1_S_30_. The addition of trace amounts of MWCNTs contributed to the dispersion of SBS particles in PPR matrix. Small and uniform SBS elastomer particles are conductive to the homogeneity of matrix and thus results in increased toughness [29]. Smaller rubber particles in the PPR matrix are tougher and more ductile than those with larger particles, because the rubbery phase with smaller size is more efficient in promoting crazing and/or shear yielding [30,31,32]. From the enlarged SEM and TEM images of C_0.05_B_0.1_S_30_ in Figure 8e,f, well-dispersed MWCNTs are mainly distributed in the interface between SBS particles and PPR matrix. Although most portion of the MWCNTs embed in the interior of the SBS particle, one end of it is tightly inserted into the PPR matrix. The interfacial interaction between SBS particles and PPR matrix is enhanced obviously with the aid of MWCNTs-bridging connection. The connection probability between SBS particles therefore increased due to the increment of the effective interaction radius of MWCNTs/SBS particles. In addition, such interface interaction helps dispersion of SBS particles in PPR matrix.

### 3.5. Glass Transition Temperatures

**Figure 9:**
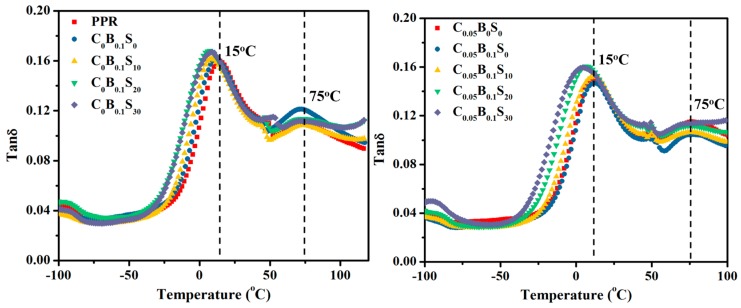
The loss factor (tanδ) of the PPR and its blends as a function of temperature.

To further investigate the toughening mechanism of PPR blends, the DMA measurement was conducted. The storage modulus curves versus temperature can be found in Figure S6 (Supporting Information). The *tan*δ of PPR and its blends are shown in Figure 9. For pure PPR, two distinct peaks can be observed. One peak at about 15 °C, corresponding to β-relaxation of PP phase, is related to the glass transition temperature (*T*_g_) of the unconstrained PPR amorphous phase, while the other peak at about 75 °C represents the α-relaxation of the PPR phase, which is related to the *T*_g_ of rigid amorphous region between PPR lamellae. It is clear that with the increase of SBS particles, the β-relaxation peak of PPR downshifts to a low temperature, along with the obviously enhanced intensity of this peak, whether the PPR blends contain binary or ternary modifiers. Some SBS molecules penetrate into the PPR matrix and form large amorphous regions, resulting in the increased motion of PPR molecules and thus the improved toughness of PPR matrix. In addition, a closer inspection revealed the β-relaxation peak of ternary modifiers/PPR blends moved to a lower temperature compared with the binary modifiers/PPR counterparts. This should be ascribed to the presence of MWCNTs.

### 3.6. Toughening Mechanism

**Figure 10:**
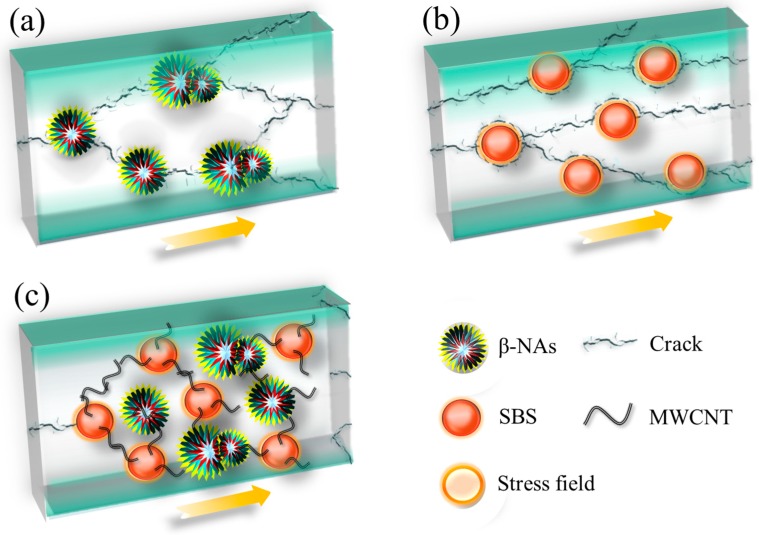
Schematic diagram of toughening mechanism of PPR blends (**a**) β-NAs/PPR; (**b**) SBS/PPR; (**c**) ternary (MWCNTs + β-NAs + SBS)/PPR.

On the basis of the analysis above, the β-NAs can induce more ductile β-form crystals to increase the toughness of the PPR matrix. The ductile β-form crystal blunted and diminished the stress intensity at a propagating crack tip. Therefore the main crack growth was restrained in the original direction and will propagate in a scattered way when encountering the β-form crystal (as shown in Figure 10a). The toughening mechanism of elastomer on plastics is usually attributed to the formation of matrix shear zone and crazing, plastic deformation, crack deflection, and particle pullout [31,33,34]. For the PPR blends containing SBS elastomer systems, plastic deformation occurs at the crack tip when the initiative microcrack encounters the SBS elastomer particles, and thus causes dis-bonding between SBS particles and PPR matrix. Therefore, some SBS elastomer particles are pulled out from the matrix under impact load (Figure 7) and the crack can be deviated from the original propagation path. This results in greater impact energy consumed and improved toughness of PPR (as shown in Figure 10b). The addition of MWCNTs alone can only increase the strength of PPR, and may be not good for the toughness. This is mainly attributed to the α-nucleation effect of MWCNTs and stress concentration derived from the existence of MWCNTs. However, the MWCNTs, acting as bridges, can effectively connect the isolated SBS elastomer particles and transfer the stress among SBS particles. Combining the positive impact of MWCNTs on the dispersion of SBS particles, large amounts of homo-dispersed SBS particles will work together to withstand the impact loads. Therefore, an obvious synergistic toughening effect is found in the ternary hybrid systems. The synergistic toughening mechanism is shown in Figure 10c. At low SBS contents, only a few SBS particles can be connected by MWCNTs due to the large distance within them. The number of the interconnected SBS particles gets increased with the increasing SBS content, resulting in more absorption of the impact energy. Therefore, the ternary modifiers (MWCNTs + β-NAs + SBS) with high SBS contents in the PPR matrix exhibit better toughening efficiency than those with low SBS contents. In addition, since the β-relaxation of PPR occurs at about 15 °C, the relatively obvious toughening enhancement of PPR blends is only observed at high temperatures (>15 °C).

## 4. Conclusions

The notched izod impact of PPR slightly improved with the addition of β-NAs due to the formation of ductile β-form crystals. Although the toughness of the PPR was significantly enhanced by combining β-NAs with SBS elastomer, the tensile strength is seriously deteriorated in the presence of SBS elastomer, especially at the high filler content. Relying on the bridge connection effect of the MWCNTs in the ternary modifiers/PPR ((MWCNTs + β-NAs + SBS)/PPR) blend, obviously synergistically reinforced toughening effect was presented. The notched izod impact strength of ternary modifiers/PPR blend with 30 wt % SBS increased from 11.3 to 58.9 kJ/m^2^; more than five-fold increment compared with pure PPR matrix, while the tensile strength retention is still above 72.2%. Moreover, the toughening efficiency is more obvious at high temperature (>15 °C) due to the β-relaxation of PP occurring at about 15 °C. These findings provide a significant guidance for fabricating high toughness PPR simultaneously with favorable tensile strength.

## Figures and Tables

**Table 1:** Sample codes and ingredients of all the Polypropylene random co-polymer (PPR) blends.

Samples	PPR (wt %)	SBS (wt %)	TMB-5 (wt %)	MWCNTs (wt %)
PPR	100	0	0	0
C_0_B_0.1_S_0_	100	0	0.1	0
C_0_B_0.1_S_10_	90	10	0.1	0
C_0_B_0.1_S_20_	80	20	0.1	0
C_0_B_0.1_S_30_	70	30	0.1	0
C_0.05_B_0_S_0_	100	0	0	0.05
C_0.05_B_0.1_S_0_	100	0	0.1	0.05
C_0.05_B_0.1_S_10_	90	10	0.1	0.05
C_0.05_B_0.1_S_20_	80	20	0.1	0.05
C_0.05_B_0.1_S_30_	70	30	0.1	0.05
C_0.05_B_0_S_30_	70	30	0	0.05

**Table 2:** Crystallization parameters of the PPR and its blends from DSC results.

Samples	*T*_c_/°C	α-form		β-form		Total *X*_c_
		*T*_m_/°C	*X*_α_	*T*_m_/°C	*X*_β_	
PPR	99.2	141.1	0.19			0.19
C_0_B_0.1_S_0_	100.9	140.6	0.15	125.5	0.09	0.24
C_0_B_0.1_S_10_	100.3	140.2	0.21	124.2	0.10	0.31
C_0_B_0.1_S_20_	98.9	139.2	0.18	123.0	0.11	0.29
C_0_B_0.1_S_30_	98.6	138.5	0.16	122.0	0.10	0.26
C_0.05_B_0_S_0_	99.7	141.1	0.21			0.21
C_0.05_B_0.1_S_0_	104.7	140.0	0.18	125.5	0.12	0.30
C_0.05_B_0.1_S_10_	101.7	139.2	0.17	124.1	0.08	0.25
C_0.05_B_0.1_S_20_	99.1	138.5	0.17	122.5	0.07	0.24
C_0.05_B_0.1_S_30_	98.1	138.2	0.16	121.1	0.06	0.22

Note: *T*_c_: crystallization temperature; *T*_m_: melting temperature; *X*c: crystallinity.

**Table 3:** The crystallization data of PPR and its blends calculated from the WAXD.

Samples	*X*_c_	*K_β_*	*X_β_*
PPR	0.35	--	--
C_0_B_0.1_S_0_	0.36	0.12	0.05
C_0_B_0.1_S_10_	0.31	0.24	0.08
C_0_B_0.1_S_20_	0.29	0.28	0.08
C_0_B_0.1_S_30_	0.28	0.31	0.09
C_0.05_B_0_S_0_	0.33	--	---
C_0.05_B_0.1_S_0_	0.35	0.26	0.09
C_0.05_B_0.1_S_10_	0.31	0.26	0.08
C_0.05_B_0.1_S_20_	0.30	0.26	0.08
C_0.05_B_0.1_S_30_	0.26	0.17	0.05

**Table 4:** The parameters of lamellar structures of PPR and its blends.

Samples	*S* (1/nm)	*L*_B_ (nm)	*L*_c_ (nm)	*L*_a_ (nm)
PPR	0.0802	12.5	4.4	8.1
C_0_B_0.1_S_0_	0.0788	12.7	4.6	8.1
C_0_B_0.1_S_10_	0.0774	12.9	4.0	8.9
C_0_B_0.1_S_20_	0.0732	13.7	4.0	9.7
C_0_B_0.1_S_30_	0.0710	14.1	3.9	10.2
C_0.05_B_0_S_0_	0.0798	12.5	4.1	8.4
C_0.05_B_0.1_S_0_	0.0785	12.7	4.4	8.3
C_0.05_B_0.1_S_10_	0.0769	13.0	4.0	9.0
C_0.05_B_0.1_S_20_	0.0703	14.2	4.3	9.9
C_0.05_B_0.1_S_30_	0.0700	14.3	3.7	10.6

## Supplementary Materials

The following are available online at http://www.mdpi.com/2073-4360/11/1/29/s1, Figure S1: GPC informations of PPR used in this study, Figure S2: SEM (a) and TEM (b and c) images of TNM5, Figure S3: Izod impact strength of PPR and its blend at various temperature (a) 20 °C, (b) 0 °C, (c) −10 °C, (d) −20 °C, Figure S4: 1D-WAXD of PPR and its blends, Figure S5: 1D-SAXS of PPR and its blends, Figure S6: The storage modulus of PPR and its blends.
