# Supplementary material for: Synergetic Toughening Effect of Carbon Nanotubes and β-Nucleating Agents on the Polypropylene Random Copolymer/Styrene-Ethylene-Butylene- Styrene Block Copolymer Blends
Source: Polymers (Basel). 2018 Dec 26;11(1):29. doi: 10.3390/polym11010029 (PMC6401747; doi:10.3390/polym11010029)
Supplement: Supplementary file 1 [file polymers-11-00029-s001.pdf]

polymers MDPI

Supporting Information

Synergetic toughening effect of carbon nanotubes and β-nucleating agents on the polypropylene random copolymer/styrene-ethylene-butylene-styrene block copolymer blends

Peng-Gang Ren[a,*], Jin Wang[a], Qian Fan[b], Song Yang[b], Zhi-Qiang Wu[b], Ding-Xiang Yan[c,*], Yan-Hui Chen[b,*]

[a]School of Materials Science and Engineering, Xi'an University of Technology, Xi'an Shaanxi 710048, People's Republic of China
[b]Department of Applied Chemistry, School of Science, and Shaanxi Key Laboratory of Macromolecular Science and Technology, Northwestern Polytechnical University, Xi'an Shaanxi 710072, People's Republic of China
[c]College of Polymer Science and Engineering, Sichuan University, Chengdu 610065, People's Republic of China
**Correspondence: rengpg@126.com (P.-G. R); yanhuichen@nwpu.edu.cn (Y.-H. C)*

2. Experimental

2.1. Materials

PPR was supplied by China Petroleum and Chemical Corporation (Nanjing, China), which has an ethylene content of 7.0 mol% and a melt flow rate of 3 g/ 10 min (230 oC, 2.16 kg), Mn = 8.5 × 104 g mol-1, Mw = 26.3 × 104 g mol-1, and polydispersity index (Mw/Mn) =3.08, detail GPC information of PPR can be obtained as following:

Cirrus GPC Sample Injection Report
Generated by: Administrator Friday, February 24, 2017 9:04 AM
Workbook: F:\Cirrus Workbooks\20140701\20140701.plw
Sample Details
Sample Name: MHL Acquired: 2/23/2017 8:28:56 PM
Batch Name: 20170224 Filename: F:\Cirrus Workbooks\20140701\20170224-0007.cgrm
Concentration: 0.10 mg/ml Injection Volume: 200.0 ul
Workbook Details
Eluent: TCB stabilised with 0.0125% BHT Flow Rate: 1.00 ml/min
Column Set: 3 x PLgel Olexis 300 x 7.5 mm Column Set Length: 0 mm
Detector: RI Temperature: 150
Analysis Using Method: 20170223
Comments:

Calibration Used: 2/22/2017 2:25:11 PM

High Limit MW RT: 15.23 mins Low Limit MW RT: 24.55 mins

High Limit MW: 6752688 Low Limit MW: 628

K: 17.5000 FRM Name:

Alpha: 0.6700 Flow Marker RT: 0.00 mins

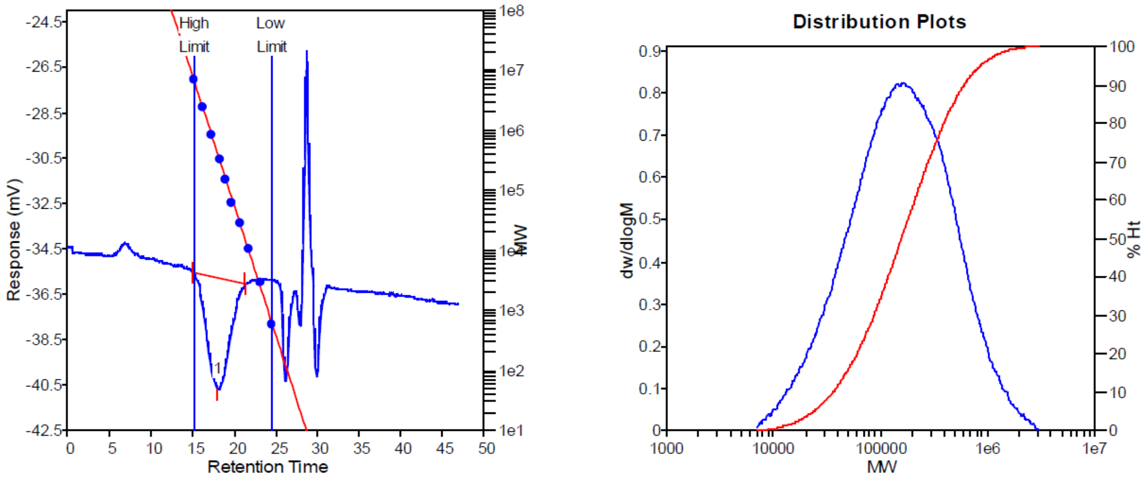

Figure S1 GPC informations of PPR used in this study

MW Averages

Peak No	Mp	Mn	Mw	Mz	Mz+1	Mv	PD
1	163330	85397	262966	630707	1101979	228673	3.07934

Processed Peaks

Peak No	Name	Start RT	Max RT	End RT	Pk Height	% Height	Area	% Area
(mins)	(mins)	(mins)	(mV)		(mV.secs)			
1		15.03	18.07	21.32	-4.91543	100	853.213	100

Peak Detection

Peak No	Type	St Detect Code	End Detect Code	Is St Mod	Is Max Mod	Is End Mod
1	0	1	1	No	No	No

Baseline Detection

No	Start RT	End RT	Start Height	End Height	Is St Mod	Is End Mod
(mins)	(mins)					
1	15.03	21.38	-35.57	-36.04	No	No

2. Experimental

2.1. Materials

Multi-wall carbon nanotube (MWCNTs, trade name: TNM5) were obtained from Chengdu Organic Chemicals Co. Ltd., Sichuan, China. Some technical parameters are sa follows: Purity:>98%, OD: 20-30nm, ID: 5-10nm, Length: 10-30μm, SSA: >110m²/g. SEM and TEM images of TNM5 can be obtained from Figure. S2.

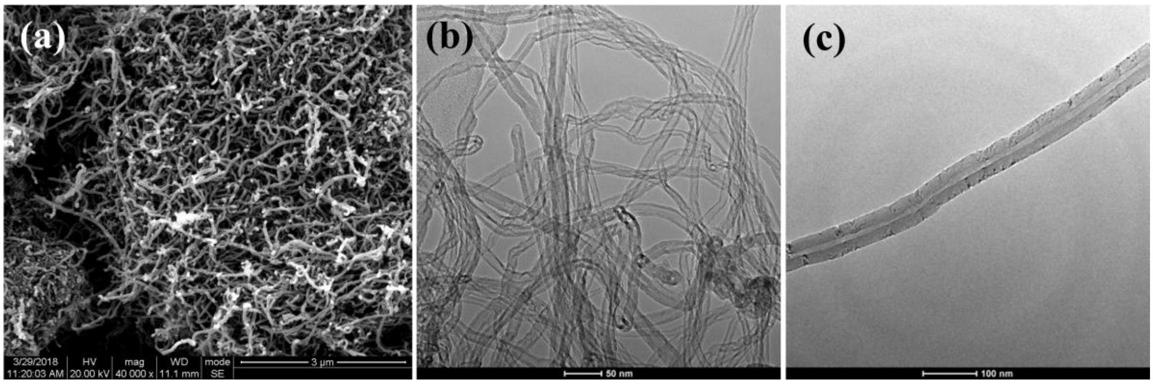

Figure S2 SEM (a) and TEM (b and c) images of TNM5

3. Results and discussion

Notched izod impact performance

The notched izod impact strength of PPR and its blends at different temperatures are displayed in Figure S3.

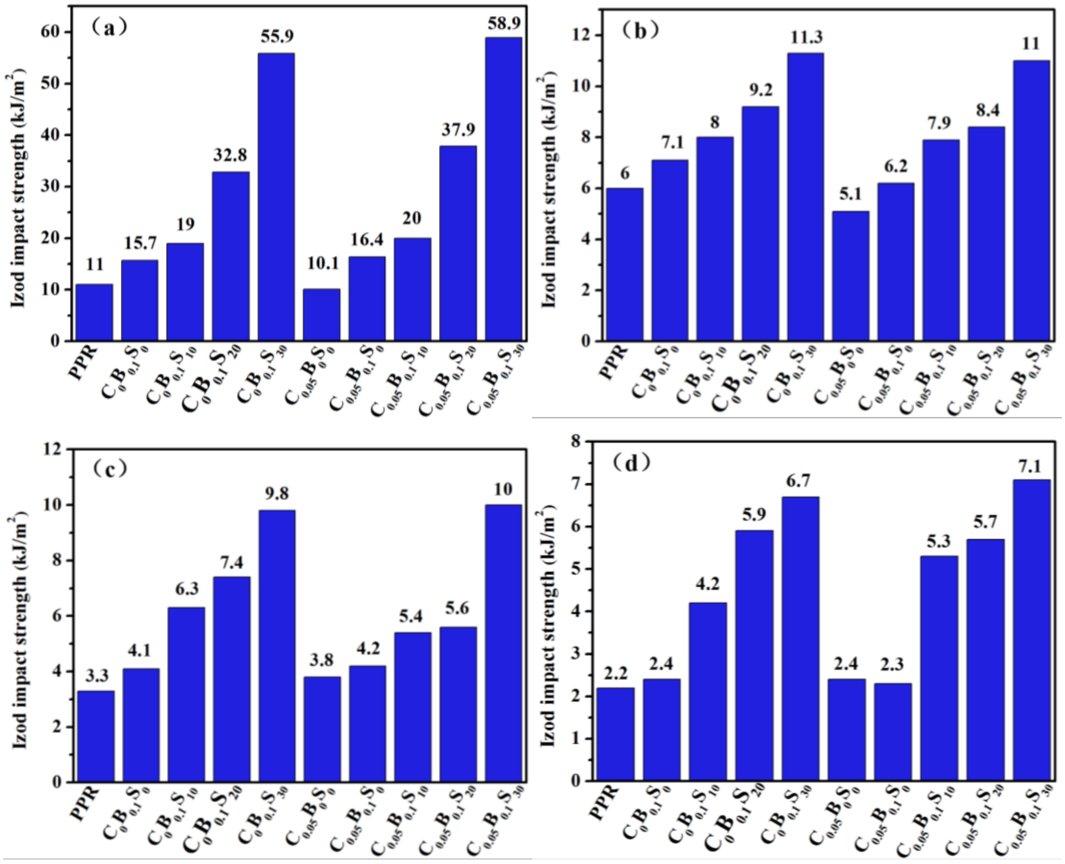

Figure S3. Izod impact strength of PPR and its blend at various temperature (a) 20°C, (b) 0°C, (c) -10°C, (d) -20°C.

Melting and crystallization behavior

The overall crystallinity (Xc), relative amount of the β-form crystal (K_{β}) and β-crystallinity (X_{β}) can be calculated from 1D-WAXD. The 1D-WAXD of PPR and its blend systems converted form 2D-WAXD patterns are shown in Figure S4.

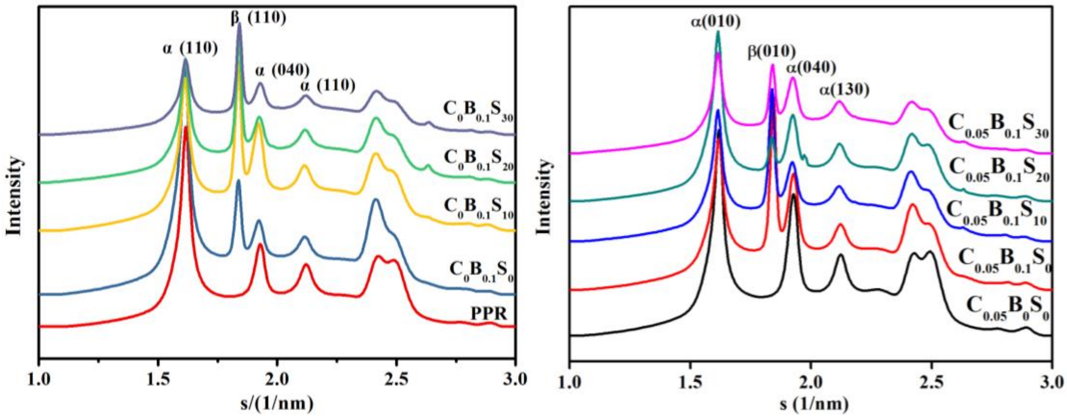

Figure S4. 1D-WAXD of PPR and its blends.

Detailed information including the long period (L_B), the thickness of crystalline lamella (L_c) and the thickness of the confined amorphous phase (L_a) can be obtained from the 1D-SAXS curves of PPR and its blends. The 1D-SAXS curves integrated from 2D-SAXS are shown in Figure S5.

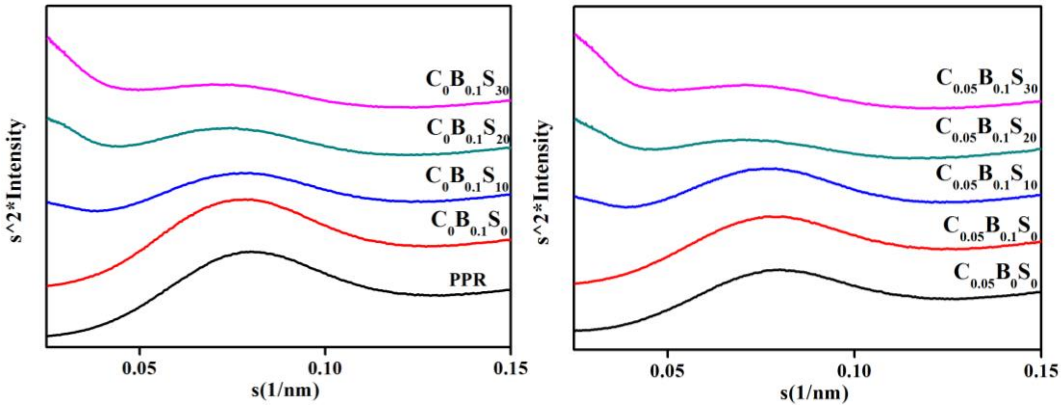

Figure S5. 1D-SAXS of PPR and its blends.

Glass transition temperatures

The storage modulus curves of PPR and its blends versus temperature are listed in Figure S6. Slightly enhanced storage modulus of PPR containing β-NAs($C_0B_{0.1}S_0$) under -100°C may be attributed to the increase in crystallinity of PPR. However, the storage modulus of PPR blends at high temperature decline with the addition of SBS particles. This implies that both the binary (β-NAs+SBS) and ternary (MWCNTs+β-NAs+SBS) displays more effective toughening for PPR under high temperature.

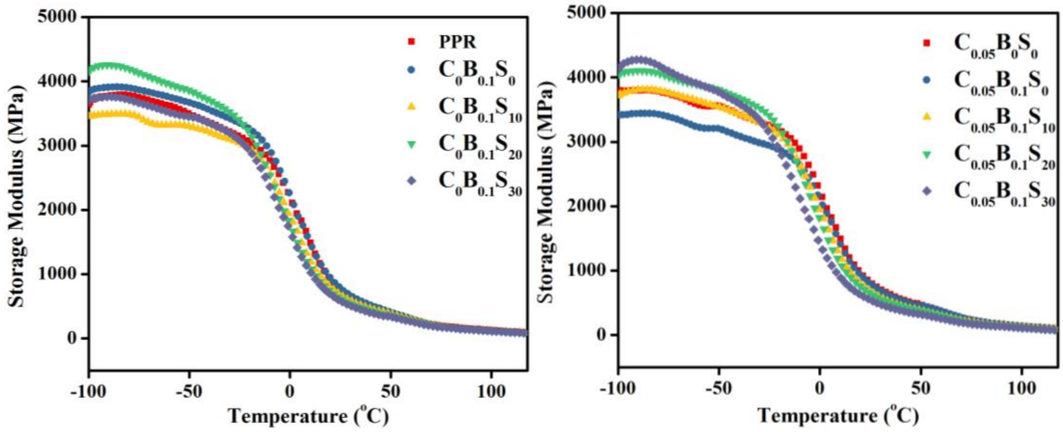

Figure S6. The storage modulus of PPR and its blends.
